# Occurrence of *Citrobacter* spp.-Associated and Non-Associated Lesions in a Stranded Loggerhead Sea Turtle (*Caretta caretta*) from Italy

**DOI:** 10.3390/pathogens15010056

**Published:** 2026-01-06

**Authors:** Filippo Fratini, Rossana Schena, Sinem Arslan, Alessandro Beneforti, Ilaria Resci, Marco Salvadori, Annunziata Romano, Luisa De Martino, Francesca Paola Nocera

**Affiliations:** 1 Department of Veterinary Sciences, University of Pisa, Viale delle Piagge, 2, 56124 Pisa, Italy; filippo.fratini@unipi.it (F.F.); a.beneforti@studenti.unipi.it (A.B.); ilaria.resci@phd.unipi.it (I.R.); 2 Department of Veterinary Medicine and Animal Production, University of Naples Federico II, 80137 Naples, Italy; rossana.schena@unina.it (R.S.); sinem.arslan@unina.it (S.A.); annunziata.romano@unina.it (A.R.); luisa.demartino@unina.it (L.D.M.); 3 Centro Veterinario Exotic Vet, Via Ulisse Dini, Gello, 157, 56017 San Giuliano Terme, Italy; exoticvet19@gmail.com

**Keywords:** *Citrobacter* spp., *Caretta caretta*, antimicrobial resistance, ESBL and MBL genes

## Abstract

The skin of turtles, particularly aquatic species, can harbor a diverse range of bacteria, including *Citrobacter* species, which are recognized as causative agents of Septicemic Cutaneous Ulcerative Disease. Consequently, turtles may act as reservoirs of pathogenic and multidrug-resistant bacteria, posing a potential public health concern. This case-based study investigated the presence of *Citrobacter* spp. in a loggerhead sea turtle (*Caretta caretta*) housed at the Livorno Aquarium, Italy. Nine swabs were collected from skin lesions (plastron, carapace, nuchal mass), the oral cavity, and the cloaca. The isolated strains were identified by MALDI-TOF MS and tested for their susceptibility to 12 antimicrobials, belonging to eight antimicrobial classes, by the disc diffusion method. Isolates were investigated genotypically for extended-spectrum-β-lactamase (ESBL) *bla_CTX−M_*, *bla_TEM_*, *bla_SHV_*, *bla_PER_*, and metallo-β-lactamase (MBL) *bla_IMP_*, *bla_OXA−48_*, *bla_VIM_*, *bla_NDM_*, *bla_GES_* genes. Biofilm production ability was also evaluated. Fifteen *Citrobacter* spp. strains were recovered from the analyzed samples. Complete resistance was recorded for ampicillin, followed by high levels of resistance to imipenem, tetracycline and piperacillin-tazobactam. Worryingly, 86.7% were classified as multidrug-resistant. The most common ESBL-genotype combination was *bla_SHV_* and *bla_PER_* genes (60%), while the most frequently detected MBL gene was *bla_NDM_* (46.7%), followed by *bla_GES_* (40%). Most isolates were classified as weak biofilm producers (80%). The findings of this study demonstrate the presence of *Citrobacter* spp., an opportunistic pathogen, with a notable prevalence of multidrug-resistant strains carrying beta-lactamase-encoding genes, in a loggerhead sea turtle in Italy, across both lesioned and healthy anatomical sites.

## 1. Introduction

The skin of turtles, especially sea turtles, represents a complex interface between the animal and the surrounding marine environment, serving as a potential ecological niche for a wide range of microorganisms [1]. Similar to other reptiles, turtles often carry enteric bacteria on their skin or shells, likely due to environmental exposure [2]. The skin microbiota of these animals frequently includes bacteria belonging to *Citrobacter* spp., particularly *Citrobacter freundii*, which are recognized as opportunistic pathogens capable of causing infections in both animals and humans [3,4].

*Citrobacter* spp. are Gram-negative, rod-shaped, non-spore forming, motile, facultative anaerobic bacilli belonging to the *Enterobacteriaceae* family. These oxidase-negative bacteria are known to generally use citrates as unique carbon source [5]. They are widespread, occurring in soil, water, sewage, food and gastrointestinal tracts of animals and humans [3,4]. Taxonomically, *Citrobacter* spp. has been described as closely related to *Escherichia coli* and *Salmonella* spp. [6,7]. Indeed, like these bacteria, *Citrobacter* spp., such as *Citrobacter freundii*, *Citroacter koseri* and *Citrobacter braakii* have been described as opportunistic pathogens, responsible for severe infections like sepsis, bacteremia, meningitis [8,9]. They have also been associated with urinary tracts infections, respiratory infections, gastrointestinal and skin infections [10,11,12]. In particular, *Citrobacter* spp. have occasionally been isolated from cutaneous lesions in marine turtles affected by septicemic cutaneous ulcerative disease (SCUD). Their role in SCUD remains unclear, as these bacteria may act either as opportunistic pathogens contributing to lesion progression and septicemia or as incidental colonizers of already compromised tissues [13].

The global spread of multidrug-resistant bacteria poses a growing threat to public and animal health [14,15]. Marine environments, including sea turtles as apex predators and bioindicators, play an important role in harboring and disseminating resistant bacteria originating from anthropogenic sources [16]. Sea turtles may act as environmental reservoirs, facilitating zoonotic transmission and the spread of resistance genes, particularly threatening vulnerable populations. In parallel, antimicrobial resistance in *Citrobacter* spp. is an increasing public health concern due to resistance determinants carried on both plasmids and chromosomes, which enhance their pathogenic potential [9]. It has been reported, overall in human medicine, that these bacteria carry numerous types of antimicrobial resistant genes, such as AmpC β-lactamases, extended-spectrum-β-lactamases (ESBLs), metallo-β-lactamases (MBLs), responsible for resistance to beta-lactam antibiotics such as carbapenems considered critically important antibiotics in medicine, and various plasmid-mediated quinolone resistance determinants [11,17,18]. Therefore, *Citrobacter* spp. is hypothesized to be a potential source for the acquisition and dissemination of antimicrobial resistant genes [19].

Currently, there is limited information regarding the prevalence of *Citrobacter* spp. in animals, particularly free-living species such as turtles, and on the spread of antimicrobial resistant strains among them. To date, the prevalence and characteristics of ESBL/MBL-producing *Citrobacter* spp. in sea turtles remains undocumented. Understanding their spread is of public health relevance, as it may indicate marine environmental contamination and the potential role of turtles as reservoirs of antimicrobial resistance determinants. This is especially relevant given the recent ban on the use of carbapenems, monobactams, and certain cephalosporin-β-lactam inhibitor combinations in veterinary medicine in Europe, as established by Commission Implementing Regulation (EU) 2022/1255 [20], which designates these critical drugs for human use only.

This case-based study aimed to characterize *Citrobacter* spp. recovered from lesions on the carapace, plastron, and skin, as well as from healthy anatomical sites (oral cavity and cloaca) of a loggerhead sea turtle (*Caretta caretta*) housed at the Livorno Aquarium, Tuscany Region, Italy. In addition, the phenotypic and genotypic antimicrobial resistance profiles of the isolates were determined, including, for the first time, the detection of ESBL- and MBL-encoding genes, together with the assessment of their biofilm-forming ability, given the established link between biofilm formation and antimicrobial resistance.

## 2. Materials and Methods

### 2.1. Ethics Statement

No animals were used in this study. Noninvasive swabs were collected from skin lesions, the oral cavity, and the cloaca in a loggerhead sea turtle undergoing diagnostic procedures as part of its clinical care at the Livorno Aquarium, Livorno, Italy. Therefore, no Institutional Review Board Statement was required, since the sampling performed in this study was carried out for diagnostic procedures as part of turtle clinical care.

### 2.2. Sample Collection

**Figure 1:**
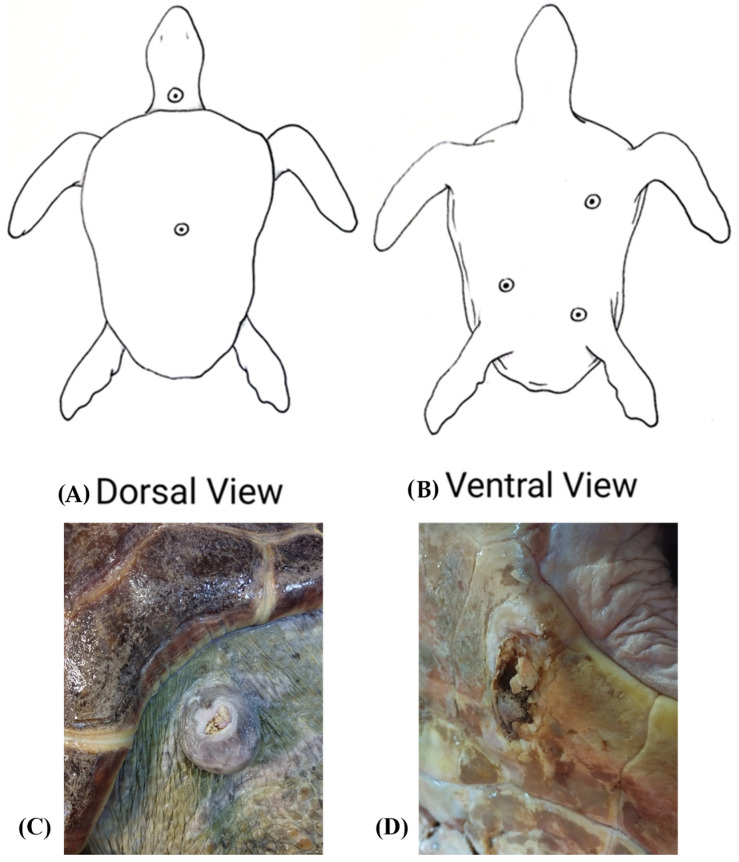
Sampled lesions in a loggerhead sea turtle (*Caretta caretta*). (**A**) Dorsal view showing the central lesion on the carapace and the ulcerated nuchal mass. (**B**) Ventral view showing the plastron lesions. (**C**) Nuchal mass. (**D**) Plastron lesion.

A loggerhead sea turtle (*Caretta caretta*) was housed at the Livorno Aquarium of the Toscana Region, Italy, for the treatment of skin lesions probably resulting from trauma, presumably caused by a collision with a boat. Sampling was performed by swabbing the skin lesions, the oral cavity and the cloaca of the loggerhead sea turtle. In total, nine swabs were collected: four from the plastron lesions, one from a central lesion on the carapace, one from an ulcerated nuchal mass, one from the oral cavity and two cloacal swabs. The sampled skin lesions are showed in Figure 1. Soon after, swabs were placed into transport medium and transferred to the Bacteriology Laboratory of the Department of Veterinary Sciences, University of Pisa, Pisa, Italy, within two hours, keeping them at 4 °C by using an ice-cooling box.

### 2.3. Citrobacter *spp.* Isolation and Identification

Once delivered in the laboratory of the Department of Veterinary Sciences of the University of Pisa, specimens were seeded on selective solid media for *Enterobacteriaceae*.

A pre-enrichment step in peptone water was performed and incubated at 37 °C for 24 h. After the overnight incubation, a loopful was taken from each pre-enrichment sample and subsequently seeded on the selective and differential solid media for *Enterobacteriaceae*, Salmonella-Shigella Agar and Brilliant Green Agar (Liofilchem, Teramo, Italy). The plates were then incubated at 37 °C for 24 h. The colonies presumptively attributable to microorganisms belonging to the *Enterobacteriaceae* family were collected with an infixation needle, seeded in Triple Sugar Iron Agar (TSI) (Thermo Fisher Scientific, Waltham, MA, USA) and finally incubated at 37° degrees for 24 h. Gas and hydrogen sulfite (H_2_S) production were highlighted. Colonies from TSI were seeded on Tryptic Soy Agar plates (Liofilchem, Teramo, Italy) to obtain pure cultures, which, after the overnight incubation, were stored in 30% glycerol at −20 °C until use.

The identification of the isolates and the evaluation of their antimicrobial resistance profiles were carried out at the Bacteriology Laboratory of the Department of Veterinary Medicine and Animal Production, University of Naples “Federico II”, Naples, Italy. Once arrived in Naples, the isolates were streaked on Mac Conkey agar plates (Liofilchem, Teramo, Italy) and, after the overnight incubation, were identified by using the Matrix-Assisted Laser Desorption/Ionization Time-of-Flight Mass Spectrometry (MALDI-TOF MS), using the MALDI Biotyper Sirius system (Bruker Daltonics Inc., Bremen, Germany). The identification procedure was performed as follows: a single bacterial colony was directly spotted onto a MALDI-TOF MS target plate. Subsequently, one microliter of α-cyano-4-hydroxycinnamic acid (HCCA) matrix solution was added to each spot and allowed to air-dry at room temperature for approximately ten minutes. The prepared target plate was then inserted into the instrument for MALDI-TOF MS analysis. The obtained spectra were analyzed and interpreted according to the manufacturer’s guidelines. In particular, based on the Bruker Biotyper scoring system, a score value > 2.0 was considered indicative of a highly probable species-level identification, a score ranging from 1.70 to 1.99 corresponded to a probable genus-level identification, and a score < 1.70 was regarded as unreliable identification. *E. coli* ATCC 25922 was used as quality control strain.

### 2.4. Evaluation of Antimicrobial Resistance Profiles in Citrobacter *spp.* Strains

The antimicrobial resistance profiles of the recovered strains were evaluated by disk diffusion method on Mueller Hinton Agar plates (Liofilchem, Teramo, Italy). All strains were tested for their susceptibility to the following 12 antimicrobials belonging to eight antimicrobial classes: amoxicillin-clavulanate (AMC 20/10 µg), ampicillin (AMP 10 µg), aztreonam (ATM 30 µg), cefalexin (CL 30 µg), ceftriaxone (CRO 30 µg), ciprofloxacin (CIP 30 µg), gentamicin (CN 10 µg), imipenem (IMI 10 µg), meropenem (MRP 10 µg), piperacillin-tazobactam (TPZ 100/10 µg), sulfamethoxazole-trimethoprim (SXT 23.75/1.25 µg), tetracycline (TE 30 µg). Strains were classified as susceptible, intermediate, and resistant to the selected antimicrobials according to the European Committee on Antimicrobial Susceptibility Testing [21] guidelines. However, for amoxicillin-clavulanate and gentamicin, the breakpoints established by the Clinical and Laboratory Standards Institute [22] were applied. Furthermore, the choice of antimicrobials also took into account those most commonly used in turtle clinical practice. *Citrobacter* spp. strains were classified as multidrug-resistant (MDR) if they exhibited resistance to at least three different antimicrobial classes, according to [23].

### 2.5. ESBL and MBL Gene Carriage in Citrobacter *spp.* Strains

DNA was extracted from the isolated *Citrobacter* spp. strains by boiling. Briefly, a few colonies of each bacterial strain were suspended in 100 µL of sterile distilled water and then boiled at 100 °C for 10 min. After the bacterial DNA were left at room temperature for 10 min and then stored at −20 °C. ESBL *bla_CTX−M_*, *bla_TEM_*, *bla_SHV_*, *bla_PER_*, and MBL *bla_IMP_*, *bla_OXA−48_*, *bla_VIM_*, *bla_NDM_*, *bla_GES_* resistance genes were detected by PCR, using specific primers purchased by Eurofins Genomics GmbH (Ebersberg, Germany) according to [24]. In-house positive- and negative control *E. coli* strains, for which both phenotypic and genomic antimicrobial resistance profiles were available, were used to validate the presence of ESBL and MBL genes. PCR experiments were performed in triplicate for each investigated gene. Primers sequences, annealing temperatures and amplicon sizes are reported in Table 1.

Analysis of the amplified products was performed using agarose gel electrophoresis. Specifically, the PCR products were separated using electrophoresis on 1.5% agarose gels prepared with 1× TBE buffer (PanReac Applichem ITW Reagents srl, Monza, Italy). Following the electrophoretic run, the gels were visualized and imaged under UV illumination.

### 2.6. Biofilm Formation Assay

The biofilm-forming ability of *Citrobacter* spp. strains was assessed using the quantitative crystal violet assay, as previously described by Stepanović et al. [25], with minor modifications, as already reported by Nocera et al. [26]. Specifically, overnight cultures grown in Brain Heart Infusion (BHI) broth (Liofilchem, Teramo, Italy) were adjusted to an optical density at 600 nm (OD_600_) of 0.2, corresponding to approximately 10^8^ CFU/mL. The standardized bacterial suspensions were diluted 1:2 in fresh BHI broth, and 200 μL aliquots were dispensed into the wells of sterile, flat-bottomed 96-well polystyrene microplates (Corning Inc., New York, NY, USA). Wells containing only sterile BHI broth served as negative controls to verify media sterility and establish background absorbance values. The plates were incubated statically at 37 °C for 24 h to allow for biofilm formation. After incubation, non-adherent cells were gently removed, and the wells were washed three times with sterile phosphate-buffered saline (PBS, pH 7.2) to eliminate planktonic bacteria. The plates were then air-dried at room temperature.

The adherent biofilms were stained with 200 μL of 1% (*w*/*v*) crystal violet solution (BioMérieux, Marcy l’Etoile, France) for 30 min at room temperature. Excess dye was removed by washing with sterile distilled water, and the plates were air-dried again. The bound crystal violet was subsequently solubilized with 200 μL of absolute ethanol (Carlo Erba Reagents srl, Milan, Italy) for 20 min at room temperature under static conditions. The absorbance of each well was measured at 570 nm using a Multiskan FC microplate reader (Thermo Fisher Scientific, Milan, Italy). All experiments were performed in triplicate for each strain (three wells per strain) and repeated three times independently. The cut-off optical density (ODc) was determined according to Stepanović et al. [25] as the mean OD of the negative control plus three standard deviations. Based on this threshold, strains were classified as follows: non-biofilm producers (OD < ODc), weak biofilm producers (ODc < OD ≤ 2 × ODc), moderate biofilm producers (2 × ODc < OD ≤ 4 × ODc), and strong biofilm producers (OD > 4 × ODc).

### 2.7. Data Curation and Descriptive Statistical Analysis

The obtained results were collected and catalogued in a Microsoft 365 Excel^TM^ spreadsheet for subsequent processing. Descriptive statistical analysis was subsequently performed to calculate the prevalence of *Citrobacter* spp., the phenotypic and genotypic antimicrobial resistance frequencies and biofilm formation ability of the recovered bacterial strains. The reported results relating to biofilm formation are presented as the mean values (±SD) of three independent experiments, and the graph was created by using Sigma Plot software (Jandl, Erkrath, Germany).

## 3. Results

### 3.1. Isolation and Identification of Recovered Citrobacter *spp*. Strains

Fifteen bacterial strains were isolated from the nine samples analyzed. Growth on TSI indicated gas production in 11 strains and H_2_S production in all 15 strains, respectively. Sub-cultivation of all isolates on MacConkey agar upon delivery to the Bacteriology Laboratory in Naples revealed lactose fermentation in all recovered strains.

**Figure 2:**
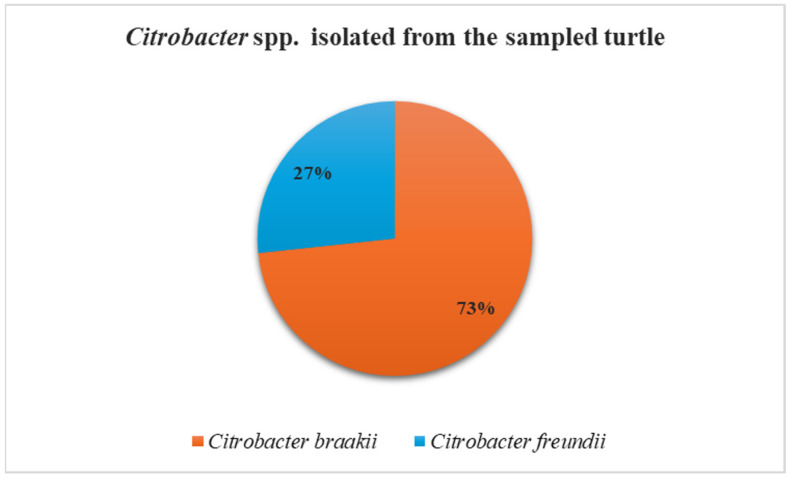
Frequency of isolation of *Citrobacter braakii* and *Citrobacter freundii* from turtle samples.

MALDI-TOF MS did not identify any isolates as *Salmonella* spp.; all recovered strains were identified as belonging to the genus *Citrobacter.* Specifically, *Citrobacter braakii* was the most frequently isolated species (73%; 11/15 strains) from all collected samples, including those associated and non-associated with lesions, followed by *Citrobacter freundii* (27%; 4/15 strains), which was recovered exclusively from non-lesioned sites, namely oral and cloacal samples (Figure 2). The MALDI-TOF MS log(score) values were ≥2.0 for all isolates, indicating highly probable species-level identification.

### 3.2. Antimicrobial Resistance Profiles of Identified Citrobacter *spp*.

**Figure 3:**
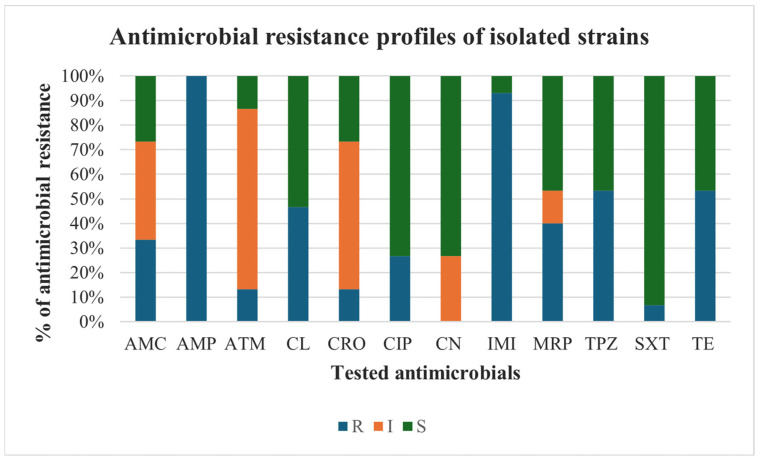
Antimicrobial resistance profiles of isolated *Citrobacter braakii* and *Citrobacter freundii* strains. Antimicrobials: AMC: amoxicillin-clavulanate; AMP: ampicillin, ATM: aztreonam; CL: cefalexin; CRO: ceftriaxone; CIP: ciprofloxacin; CN: gentamicin; IMI: imipenem; MRP: meropenem; TPZ: piperacillin-tazobactam; SXT: sulfamethoxazole-trimethoprim; TE: tetracycline. The tested strains were classified as resistant (R), intermediate (I) or susceptible (S) to tested antimicrobials.

The evaluation of the antimicrobial resistance profiles of the recovered *Citrobacter braakii* and *Citrobacter freundii* revealed interesting results for the tested antimicrobials (Figure 3). Specifically, complete resistance was recorded for ampicillin. High resistance frequencies were also observed for imipenem (93.3%), followed by tetracycline and piperacillin-tazobactam both with a value of 53.3%, cefalexin (46.7%), and meropenem (40%). Conversely, the highest levels of susceptibility were recorded for sulfamethoxazole-trimethoprim (93.3%), followed by ciprofloxacin and gentamicin (both at 73.3%). High percentages of intermediate susceptibility were noted for aztreonam (73.4%) and ceftriaxone (60%). Worryingly, 86.7% (13/15) of the strains were MDR, comprising 10 *Citrobacter braakii* and 3 *Citrobacter freundii* strains.

### 3.3. ESBL and MBL Determinants in Citrobacter *spp*.

In the investigated *Citrobacter braakii* and *Citrobacter freundii* strains, ESBL genotypic resistance was driven by *bla_PER_* and *bla_SHV_*, both of which were detected in 73.3% (11/15) of the strains. Furthermore, the *bla_PER_* and *bla_SHV_* genes were found in association in nine strains (60%). Among the studied MBL-genes, *bla_NDM_* was the most frequently detected gene (46.7%; 7/15), followed by *bla_GES_* (40%; 6/15 strains), and *bla_VIM_* (13.3%; 2/15 strains). Only in two strains (13.3%), both identified as *Citrobacter freundii*, carried the two MBL-genes *bla_NDM_* and *bla_VIM_* together. Moreover, four *Citrobacter braakii* strains co-carried ESBL *bla_SHV_*, *bla_PER_* genes and MBL *bla_NDM_* gene. Two *Citrobacter freundii* strains were positive to the detection of ESBL *bla_SHV_* gene and MBL *bla_NDM_* and *bla_VIM_* genes. Therefore, an interesting finding was the concordance between the ESBL and MBL phenotypes and genotypes across the screened *Citrobacter braakii* and *Citrobacter freundii* strains, as shown in Table 2.

### 3.4. Biofilm Formation Ability of Citrobacter braakii and Citrobacter freundii Strains

**Figure 4:**
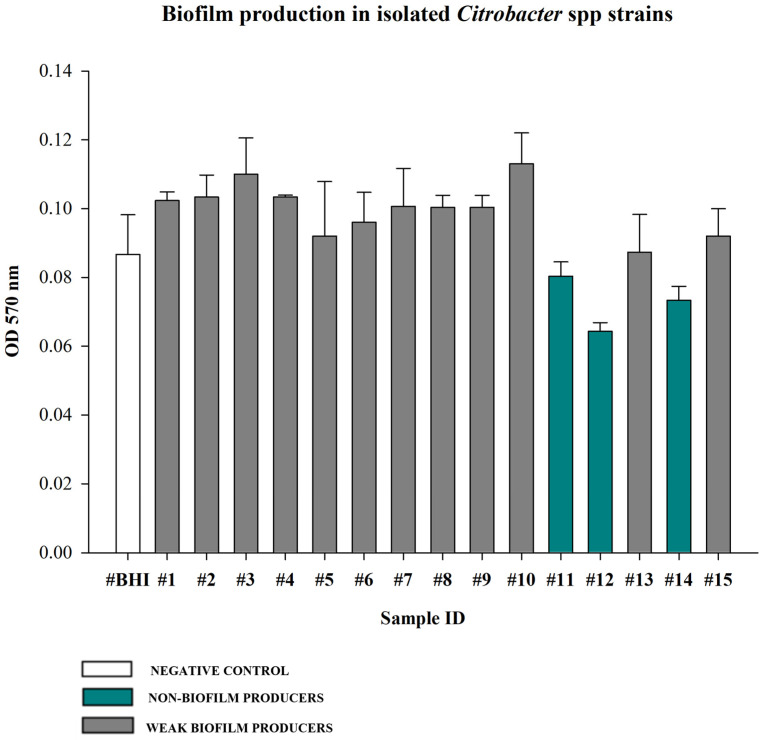
Biofilm producing ability of isolated *Citrobacter* spp. strains assessed by crystal violet quantitative assay. BHI broth was used as negative control. Biofilm production ability was determined using the mean OD 570 nm of the negative control (0.09). Values <0.09 were considered non-biofilm producers (20%), values between 0.09 and 0.18 (2× the negative control value 0.09) were considered weak producers (80%). Values between 0.18 and 0.36 (2× and 4× the negative control mean) were categorized as moderate producers (0%), and values >0.36 (4× the negative control mean) were strong producers (0%). Each bar represents the mean result of three biological experiments. Error bars indicate the standard deviation.

The recovered *Citrobacter braakii* and *Citrobacter freundii* strains were evaluated for their ability to produce biofilm (Figure 4). Most isolates were classified as weak biofilm producers (80%; 12/15 strains), while the remaining three strains (20%) were identified as non-biofilm producers. No moderate and strong producers were identified.

Furthermore, the evaluation of the biofilm producing capability in relation to the multidrug resistance and ESBL/MBL gene carriage revealed that all strains categorized as the weak biofilm producers were MDR, with the single exception of strain n. 8 (*Citrobacter braakii*). Notable, among the two non-biofilm producers, the n. 14, identified as *Citrobacter freundii*, did not show an MDR profile, but co-carried both ESBL (*bla_SHV_*) and MBL (*bla_NDM_* and *bla_VIM_*) genes.

## 4. Discussion

This study offers descriptive evidence on the occurrence and antimicrobial resistance profiles of *Citrobacter* spp. isolated from a loggerhead sea turtle (*Caretta caretta*) housed at the Livorno Aquarium (Italy). Although *Citrobacter* species are recognized as opportunistic pathogens commonly associated with the intestinal microbiota of animals and humans, their detection in external anatomical sites, such as skin lesions, the oral cavity, and the cloaca highlights their ability to colonize multiple ecological niches beyond the gut, including marine environments [9,27,28,29]. To the best of our knowledge, this is the first study to report *Citrobacter braakii* and *Citrobacter freundii* carrying ESBL and MBL genes in a sea turtle from the Mediterranean region.

*Citrobacter braakii* was the most frequently isolated species (73%) and was recovered from both lesioned and non-lesioned sites across all sampled anatomical locations. In contrast, *Citrobacter freundii* (27%) was isolated exclusively from non-lesioned sites, specifically the oral cavity and cloaca. These descriptive preliminary observations, while constrained by the single-host nature of this study, provide initial insights into the distribution of *Citrobacter* spp. in relation to lesion presence and anatomical site. The predominance of *Citrobacter braakii* among the recovered isolates is consistent with previous reports identifying this species as an emerging environmental and clinical opportunist, able to persist in aquatic ecosystems and humid environments for extended periods [29,30]. Moreover, Pasquali et al. [31] reported the isolation of *Citrobacter braakii* from fermented foods of animal origin and from the processing environments of two artisanal facilities producing soft cheese and salami, respectively, emphasizing the potential role of this emerging bacterium as a food-borne pathogen. Additionally, several studies have highlighted the frequent misidentification by biochemical systems of *Citrobacter braakii* as *Citrobacter freundii* or other *Enterobacteriaceae* members, which may have contributed to an underestimation of its occurrence and clinical relevance as an opportunistic pathogen [9,32,33]. In this study, bacterial identification was carried out using MALDI-TOF MS, which enabled the identification of *Citrobacter braakii* with a log(score) > 2.0, indicating reliable species-level identification. MALDI-TOF MS is an accurate and cost-effective method for bacterial identification, offering more accurate and faster results than traditional systems [34]. Crucially, it allowed *Citrobacter braakii* to be correctly distinguished from *Citrobacter freundii*.

In this study, the detection of *Citrobacter freundii* exclusively in the oral and cloacal swabs suggests that this species may be more closely associated with the enteric or mucosal microbiota rather than external lesions. Previous studies have reported that *Citrobacter freundii* is commonly found in the intestinal tract of animals and humans and in clinical samples such as feces and cloacal swabs, highlighting its role as an opportunistic and commensal pathogen of mucosal environments [3,11,35,36]. Furthermore, it is worth noting that *Citrobacter freundii* has been recognized as a nosocomial pathogen in both humans and companion animals, responsible for severe infections, particularly in immunocompromised patients [30,37]. These infections include bacteremia, urinary tract infections, pneumonia, and bloodstream infections. Therefore, the occurrence of these bacteria in a rehabilitated marine turtle likely reflects environmental exposure, possibly linked to anthropogenic contamination, given that *Citrobacter* spp. are often found in sewage and wastewater discharges reaching coastal ecosystems [4]. It should be noted, however, that the use of selective media, effective for the isolation of *Citrobacter* spp., may have introduced a methodological bias by potentially missing other clinically relevant bacteria within the samples.

Currently, *Citrobacter* species have become a significant global concern due to their increasing resistance to antimicrobials and ability to harbor, acquire and spread antimicrobial resistance determinants [35,38]. However, information on the spread of antimicrobial-resistant strains in animals, especially free-living and aquatic ones, is still scarce. In this study, the antimicrobial susceptibility results revealed widespread resistance among the recovered isolates, with 86.7% of the isolated strains being MDR, recovered from both lesioned and healthy anatomical sites. Of particular concern are the total resistance to ampicillin (100%) and the high frequency of resistance to imipenem (93.3%), piperacillin-tazobactam (53.3%), cefalexin (46.7%) and meropenem (40%). The observed resistance to carbapenems (imipenem and meropenem) is especially noteworthy, considering that these agents are classified by the World Health Organization as “critically important” antimicrobials for human health and, consequently, are authorized only for human use [20,39]. The high resistance levels recorded in this study to beta-lactams may be attributed to either the environmental persistence of resistant strains or the selective pressure exerted by anthropogenic antimicrobial residues introduced into the marine ecosystem. Our results agree with those of other studies, which reported the isolation of MDR *Citrobacter* species among strains of both animal and human origin [11,35,37,38,40]. Ahmed et al. [35] reported the isolation of 60% MDR *Citrobacter freundii* strains from domestic duck in Bangladesh. However, while high resistance levels were observed for antimicrobials such as ampicillin, tetracycline, and cephalexin in this study, aligning with the report by Ahmed et al. [35], we found contrasting results for other antimicrobials. Specifically, while Ahmed et al. [35] reported high resistance rates (ranging from 20% to 96%) to sulfamethoxazole-trimethoprim (cotrimoxazole), cephalexin, ciprofloxacin, and gentamicin, our *Citrobacter* spp. strains demonstrated good susceptibility to these antimicrobial agents. Moreover, a Japanese study involving *Citrobacter* spp. isolated from owned pets demonstrated lower resistance rates to several tested antimicrobials, including tetracycline and sulfamethoxazole-trimethoprim, and exhibited complete susceptibility to meropenem [37]. It is interesting to note that this study aligns with human reports describing the isolation of MDR *Citrobacter* species showing high levels of resistance to penicillin, cephalosporins, carbapenems, and lower values of resistance to quinolones, aminoglycosides, and sulfonamides [11,41].

The detection of both ESBL and MBL genes in *Citrobacter braakii* and *Citrobacter freundii* might lend further support to the hypothesis that sea turtles could potentially act as reservoirs and disseminators of resistance determinants. This finding is particularly relevant for *Citrobacter* spp., as these bacteria have been suggested to play a key role in bacterial evolution due to their ability to accumulate antimicrobial resistance determinants. Primarily characterized as low-virulence, commensal, and opportunistic bacteria, *Citrobacter* spp. may possess the capacity to persist in host populations for extended periods, thereby facilitating the spread of resistance [42]. Resistance to beta-lactams, especially cephalosporins and carbapenems, is largely attributed to the successful acquisition of resistance genes via horizontal gene transfer in *Citrobacter* spp. isolates [42]. Research focusing on carbapenem-resistant *Citrobacter* spp. consistently highlights Inc family plasmids as the primary vectors responsible for carrying carbapenem resistance genes, especially *bla_NDM_* and *bla_VIM_* [43,44,45]. In particular, the detection of *bla_NDM_*, *bla_GES_*, and *bla_VIM_* highlights the presence of carbapenemase-encoding genes that confer resistance not only to carbapenems but also to a wide range of β-lactam antibiotics, even when associated with beta-lactam inhibitors [46]. In this study the co-carriage of ESBL *bla_PER_* and *bla_SHV_* genes and MBL *bla_NDM_*, *bla_GES_* and *bla_VIM_* genes with different associations were observed in the screened *Citrobacter braakii* and *Citrobacter freundii* strains. This result is particularly alarming, as such combinations have been associated with enhanced resistance phenotypes and increased potential for horizontal gene transfer [42]. Specifically, the co-existence of *bla_NDM_*, *bla_VIM_* and *bla_SHV_* found in the investigated *Citrobacter* spp. strains has been reported in other studies [22,47,48]. However, unlike other reports, here *bla_TEM_* and bla_CTX-M_ genes were not detected, which have been frequently identified in *Citrobacter* spp. strains of human origin [45,49].

Moreover, the co-carriage of both ESL and MBL genes in both *Citrobacter braakii* and *Citrobacter freundii* strains might imply worrying clinic and therapeutic consequences, underscoring the necessity to enhance surveillance programs. Furthermore, although several isolates shared similar resistance profiles, the absence of high-resolution molecular typing, such as Whole Genome Sequencing (WGS) or Multilocus Sequence Typing (MLST), precludes the definitive confirmation of their clonal identity, leaving the exact transmission pathways yet to be fully elucidated.

The observed phenotypic variability in biofilm formation, with the majority of strains being weak biofilm producers (80%) but with 20% classified as non-biofilm producers, suggests potential differences in their adherence and persistence capabilities under in vitro conditions.

While strong biofilm formation is often correlated with increased antimicrobial resistance [50], our results highlight a more complex interplay between phenotype and genotype. In this study, nearly all biofilm-forming strains were MDR, confirming the frequent clustering of resistance mechanisms with virulence and persistence factors [50]. In marine environments, biofilms are crucial for bacterial adhesion to biotic and abiotic surfaces, such as turtle skin, shells, or debris. This persistence not only promotes bacterial survival but also facilitates the maintenance and potential dissemination of resistance genes within microbial communities [51]. A particularly interesting finding of the present study involved the isolation of a non-biofilm-producing and non-MDR strain that simultaneously carried the *bla_SHV_*, *bla_NDM_* and *bla_VIM_* genes, encoding for ESBLs and carbapenemases, respectively. This result emphasizes that even seemingly less problematic strains (non-MDR and non-biofilm-forming) can still pose a severe risk as “silent reservoirs,” capable of disseminating clinically crucial beta-lactamase-encoding genes via plasmid transfer.

It is important to acknowledge that the descriptive evidence provided in this study is derived from a single clinical case, which represents a limitation for the epidemiological interpretation of the findings across the wider *Caretta caretta* population. Furthermore, the lack of Minimum Inhibitory Concentration (MIC) determination for the tested antimicrobials constitutes another limitation of this work, as antimicrobial susceptibility was assessed solely by the disk diffusion method. Future investigations integrating MIC determination and WGS would allow for a more accurate interpretation of resistance levels and the potential environmental dissemination of ESBL and MBL determinants, thereby strengthening the One Health implications. Nevertheless, the isolation of MDR, ESBL-, and MBL-producing *Citrobacter* spp. in a sea turtle may serve as an indicator of potential environmental contamination within the One Health framework, a concern further supported by recent research in the Mediterranean Sea, which has established a strong correlation between the abundance of beta-lactamase genes (*bla_TEM_*, *bla_VIM_*) and eutrophic conditions. These findings suggest that anthropogenic nutrient enrichment may act as a primary driver for the propagation of antibiotic resistance genes [52].

## 5. Conclusions

In conclusion, this study reports, for the first time, the emergence of MDR *Citrobacter* spp. harboring ESBL and MBL genes in a loggerhead sea turtle. Despite the limitations inherent in a single-animal study, these results underscore the need for broader surveillance. Future research should adopt integrated approaches, including WGS to characterize resistance genes and transmission pathways, and a One Health framework with paired environmental sampling (water, substrate, and food) in rehabilitation facilities to identify potential reservoirs. Moreover, comparative and longitudinal studies involving free-living and rehabilitated sea turtles are needed to assess prevalence, ecological dynamics, and to distinguish transient carriage from persistent colonization of antimicrobial-resistant bacteria in the marine ecosystems.

## Figures and Tables

**Table 1:** Primers sequences, annealing temperatures and amplicon sized of tested ESBL and MBL genes.

Gene	Primer Sequence (5′-3′)	Annealing Temperature (°C)	Amplicon Size (bp)
***bla_SHV_***	F: ATGCGTTATATTCGCCTGTG R: TGCTTTGTTATTCGGGCCAA	52	747
***bla_IMP_***	F: ACAYGGYTTRGTDGTKCTTG R: GGTTTAAYAAARCAACCACC	58	387
***bla_NDM_***	F: ACTTGGCCTTGCTGTCCTT R: CATTAGCCGCTGCATTGAT	52	603
***bla_OXA-48_***	F: ATGCGTGTATTAGCCTTATCG R: CATCCTTAACCACGCCCAAATC	58	265
***bla_GES_***	F: CTGGCAGGGATCGCTCACTC R: TTCCGATCAGCCACCTCTCA	55	864
***bla_VIM_***	F: TGTCCGTGATGGTGATGAGT R: ATTCAGCCAGATCGGCATC	60	437
***bla_CTX-M_***	F: ATGTGCAGYACCAGTAARGTKATGGC R: TGGGTRAARTARGTSACCAGAAYCAGCGG	58	593
***bla_TEM_***	F: TCGCCGCATACACTATTCTCAGAATGA R: ACGCTCACCGGCTCCAGATTTAT	60	445
***bla_PER_***	F: AGTGTGGGGGCCTGACGAT R: GCAACCTGCGCAATRATAGCTT	56	725

**Table 2:** Correlation between phenotypic and genotypic detection of ESBL and MBL resistance in *Citrobacter* spp. isolates.

			ESBL-Encoding Genes				MBL-Encoding Genes				
ID	Identified Species	Phenotypic Resistance Profiles	*bla_PER_*	*bla_SHV_*	*bla_TEM_*	*bla_CTX-M_*	*bla_VIM_*	*bla_GES_*	*bla_NDM_*	*bla_OXA-48_*	*bla_IMP_*
**1**	*Citrobacter freundii*	AMP, CL, IMI, MRP						X			
**2**	*Citrobacter braakii*	AMP, IMI, MRP, SXT, TE							X		
**3**	*Citrobacter braakii*	AMC, ATM, IMI, MRP, TPZ, TE		X				X			
**4**	*Citrobacter braakii*	AMP, CL, CIP, IMI, TE	X								
**5**	*Citrobacter braakii*	AMC, AMP, IMI, TPZ, TE	X	X					X		
**6**	*Citrobacter braakii*	AMC, AMP, ATM, CL, IMI, MRP, TPZ	X	X					X		
**7**	*Citrobacter freundii*	AMP, CL, CRO, IMI, TPZ	X	X			X		X		
**8**	*Citrobacter braakii*	AMC, AMP, CL	X	X							
**9**	*Citrobacter braakii*	AMP, CL, CRO, IMI, TPZ, TE	X	X				X			
**10**	*Citrobacter braakii*	AMC, AMP, IMI, TPZ, TE	X	X				X			
**11**	*Citrobacter braakii*	AMP, CL, CIP, IMI, TPZ	X	X					X		
**12**	*Citrobacter freundii*	AMP, IMI, TPZ, TE	X	X				X			
**13**	*Citrobacter braakii*	AMP, CIP, IMI	X	X					X		
**14**	*Citrobacter freundii*	AMP, IMI, MRP		X			X		X		
**15**	*Citrobacter braakii*	AMP, IMI, MRP, TE	X					X			

**Legend:** “X” denotes the presence of the ESBL- and MBL-encoding genes in the investigated *Citrobacter* spp. isolates.

## Data Availability

The data supporting the findings of this study are available within this article. Further inquiries can be directed to the corresponding author.
